# Challenges and Perspectives of Polyhydroxyalkanoate Production From Microalgae/Cyanobacteria and Bacteria as Microbial Factories: An Assessment of Hybrid Biological System

**DOI:** 10.3389/fbioe.2021.624885

**Published:** 2021-02-19

**Authors:** Rukhsar Afreen, Shivani Tyagi, Gajendra Pratap Singh, Mamtesh Singh

**Affiliations:** ^1^Department of Zoology, Gargi College, University of Delhi, New Delhi, India; ^2^Mathematical Sciences and Interdisciplinary Research Lab (Math Sci Int R-Lab), School of Computational and Integrative Sciences, Jawaharlal Nehru University, New Delhi, India

**Keywords:** polyhydroxyalkanoates, photoautotrophic, heterotrophic, mixotrophy, hybrid biological system, two-module system, petri net

## Abstract

Polyhydroxyalkanoates (PHAs) are the biopolymer of choice if we look for a substitute of petroleum-based non-biodegradable plastics. Microbial production of PHAs as carbon reserves has been studied for decades and PHAs are gaining attention for a wide range of applications in various fields. Still, their uneconomical production is the major concern largely attributed to high cost of organic substrates for PHA producing heterotrophic bacteria. Therefore, microalgae/cyanobacteria, being photoautotrophic, prove to have an edge over heterotrophic bacteria. They have minimal metabolic requirements, such as inorganic nutrients (CO_2_, N, P, etc.) and light, and they can survive under adverse environmental conditions. PHA production under photoautotrophic conditions has been reported from cyanobacteria, the only candidate among prokaryotes, and few of the eukaryotic microalgae. However, an efficient cultivation system is still required for photoautotrophic PHA production to overcome the limitations associated with (1) stringent management of closed photobioreactors and (2) optimization of monoculture in open pond culture. Thus, a hybrid system is a necessity, involving the participation of microalgae/cyanobacteria and bacteria, i.e., both photoautotrophic and heterotrophic components having mutual interactive benefits for each other under different cultivation regime, e.g., mixotrophic, successive two modules, consortium based, etc. Along with this, further strategies like optimization of culture conditions (N, P, light exposure, CO_2_ dynamics, etc.), bioengineering, efficient downstream processes, and the application of mathematical/network modeling of metabolic pathways to improve PHA production are the key areas discussed here. Conclusively, this review aims to critically analyze cyanobacteria as PHA producers and proposes economically sustainable production of PHA from microbial autotrophs and heterotrophs in “hybrid biological system.”

## Introduction

**FIGURE 1:**
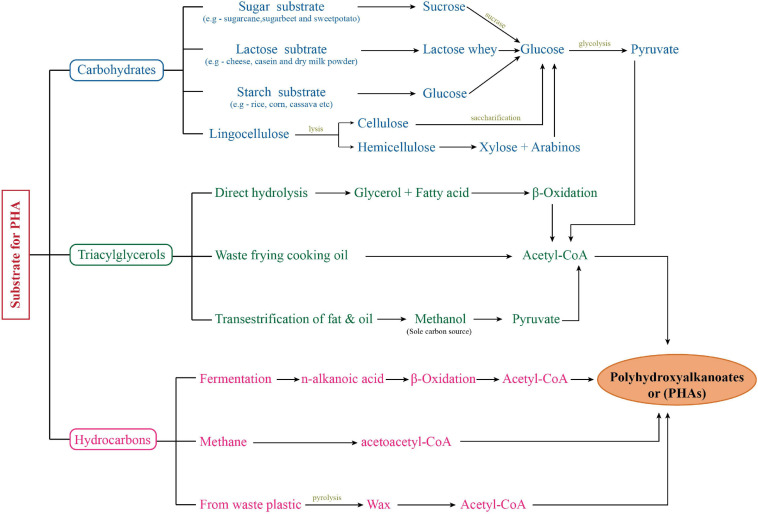
Flow chart showing PHA production from diverse substrates.

Fossil fuel depletion and non-biodegradability of petro-chemical based plastics has created a scenario where we critically need an alternative. Petroleum based plastic has been extensively used worldwide because of their high durability and inexpensive production. But their persistence due to non-biodegradable nature has raised certain serious environmental concerns. Biodegradable plastics are the best “eco-friendly” alternatives of petrochemical based synthetic plastic for protection and sustainable development of the environment (Gill, 2014; Shen et al., 2020). Polyhydroxyalkanoates (PHAs) are the best candidates for this. These are the polymers of hydroxyacids produced by number of microorganisms including bacteria. PHAs have similar properties to petroleum derived synthetic plastics like polypropylene (PP) and it is 100% biodegradable in environment (Singh et al., 2015; Sharma et al., 2020). Bacteria belonging to a number of genus are reported to have PHA producing abilities. Out of these, *Cupriavidus*, *Pseudomonas*, *Bacillus*, and recombinant *Escherichia coli* are majorly used for PHA production through microbial fermentation (Singh et al., 2009; Akdoǧan and Çelik, 2018; Brojanigo et al., 2020; Kalia et al., 2021). For PHA production, these organisms were able to utilize various substrates including pure (carbohydrates, fatty acids) and agro-industrial biowaste (Pea Shells, Sweet Potato, Whey, Beet molasses) as substrates (Figure 1; Singh et al., 2009, 2015; Kumar et al., 2013; Amaro et al., 2019; Bhatia et al., 2021; Kalia et al., 2021). But PHA production from these heterotrophic bacteria has been quite expensive which is due to the cost of carbon (C) substrates. Approximately 45% of the production cost is utilized in substrate procurement. Biowastes have been used as an effective resource and have helped substantially in cost reduction of PHA production process (Singh et al., 2015). But still there is a big economical gap in petroleum-based plastic and bioplastic. Autotrophic microorganisms provide a good alternative for economical PHA production having an edge over heterotrophic bacteria in certain aspects related to bioprocess development. Photoautotrophic organisms, e.g., cyanobacteria (collectively referred to “microalgae” along with eukaryotic microalgae in this review), are emerging as a strong contender for this purpose. Microalgae are an extremely diverse group and still have lots of opportunities unexplored (Borowitzka, 2013).

They present a strong candidature as microbial factory for various bioproducts due to their high growth rate, minimal nutrient requirements, low fresh water requirement, and high photosynthetic efficiency through which they can convert inorganic nutrients (CO_2_) into organic reserves, utilizing light as energy source. The organic reserves produced during photosynthesis can then be further processed to PHA in these cells itself or can be used as feed for other microorganisms. However, autotrophic cultivation also possesses its limitations such as (1) stringent management of closed photobioreactors and (2) optimization of monoculture in open pond culture. Alternatively, bioprocess in which both autotrophic and heterotrophic microorganisms/cultivation can be used may utilize the resources optimally and helps in improving the economy of bioprocess. Thus, in this review we will discuss cyanobacteria as PHA-producing microbial factories, their potential, and challenges and analyze the perspectives of adopting a hybrid system involving both photoautotrophic and heterotrophic components so that their interactive relationship benefits the bioprocess system. Such hybrid system can be exploited under different cultivation regime, e.g., mixotrophic, successive two modules, consortium based, etc. PHA production from cyanobacteria has been reported to be affected by factors like culture conditions: N, P, light exposure, CO_2_ dynamics, etc. In addition to that other factors like two-stage (growth and PHA accumulation) processes, metabolic inhibitors for other pathways and bioengineering have also been reported to have a positive impact on PHA production (Sharma et al., 2007; Bhati and Mallick, 2012, 2015, 2016; Kamravamanesh et al., 2017; Troschl et al., 2018). A new promisingly arising field is the application of mathematical/network modeling in this field which has increasingly been adopted to improve PHA production (Novak et al., 2015; Pillai et al., 2019). Here, we propose another tool “Petri Net” and its application in metabolic pathway or bioprocess modeling to improve PHA production.

## Microalgae as PHA Microbial Factory

Considered among the fastest growing autotrophic organisms, microalgae are the global primary biomass producers having the ability to fix atmospheric CO_2_ utilizing solar energy. With the ability to utilize minimal or readily available material, i.e., inorganic nutrients (CO_2_), water, sunlight, wastewater, and having high photosynthetic efficiency, they are the strong candidate as “microbial factory” for the production of various bioproducts (Rahman et al., 2013, 2014; Singh and Mallick, 2017). This ability of microalgae to grow in adverse environment is demonstrated in different studies reporting their cultivation in urban wastewater, swine waste, dairy manure, cattle residues, and poultry litter (PL) (Olguín et al., 2003; Mulbry et al., 2008; Ansari and Fatima, 2019). A vast variety of high value natural products, e.g., pigments, carotenoids, proteins, enzymes, sugars, fatty acids, polysaccharides, phycobilins, sterols, vitamins, and many other unusual bioactive compounds, are produced by microalgae (Olaizola and Huntley, 2003; Ratledge, 2004; Singh et al., 2005; Mendes et al., 2009; Borowitzka, 2013; Kamravamanesh et al., 2018b). They don’t need specialized fertile land and large freshwater supply and can be grown on ponds. Thus, microalgal biomass is ideal for PHA production and provides a low-cost economical bioprocess system (Rahman et al., 2014). Among them, only few eukaryotic algae are reported for PHA production, e.g., *Chlorella* (Roja et al., 2019). But cyanobacteria are taking the lead in this field (Carpine et al., 2015, 2017). Cyanobacteria are one of the largest bacterial group which are aquatic and photosynthetic known to be one of the oldest microorganisms and first photoautotrophic on earth. Thus, they played a vital role in the evolution of life creating an oxygen-rich environment and still are contributing to the carbon/oxygen cycle globally. Presence of phycocyanin pigment in them imparts them the characteristic blue–green color, and thus, they are known as blue–green algae. Fermentative PHA production is well known from heterotrophic bacteria. Similarly, most cyanobacteria also have the potential, although species specific even within same genera, to produce PHAs as intracellular carbon reserve. Like in heterotrophic bacteria, here also PHA is accumulated under nutrient (e.g., nitrogen and phosphorus) limiting conditions (Kaewbai-ngam et al., 2016). Such studies have reported the PHA production being influenced by various factors, e.g., nutrient availability, culture cultivation parameters (pH, light-dark cycles, temperature, gas/CO_2_ flow), growth phase, and stationary phase duration (Sharma and Mallick, 2005a,b; Panda et al., 2006; Wagner et al., 2016; Troschl et al., 2017, 2018). In addition to this, microalgae can achieve wastewater remediation process along with PHA production through the assimilation of phosphorus and nitrogen as growth nutrients (Rahman et al., 2013). It is interesting to note that the microalgae grown in closed laboratory photobioreactor, open ponds, or in wastewater can achieve higher biomass economically. Thus, in addition to using this biomass as microbial factory for PHA production, this can also be harvested and processed for further utilization as substrate by other heterotrophic bacteria (Rahman et al., 2014). Similar to heterotrophic bacteria, along with PHAs other polymer reserves have also been reported in cyanobacteria, glycogen being the most significant of them (Kumar and Kim, 2018). It is interesting to note that in 1966 the first report of PHA production by cyanobacteria was documented for heterotrophic PHA production in a *Chlorogloea fritschii* with acetate as C-source; however, photoautotrophic PHA production was soon reported thereafter in 1971 in *Gloeocapsa* strain 6501 (Kamravamanesh et al., 2018a). Mixotrophy and chemoheterotrophy cultivation, favored over photoautotrophy, have also been documented in various studies (Wu et al., 2001; Sudesh et al., 2002; Sharma and Mallick, 2005b; Panda et al., 2006; Wagner et al., 2016).

## PHA Biosynthesis

Polyhydroxyalkanoates production under heterotrophic microbial fermentative process has been explained quite extensively with number of researchers in this field. It is well known that the classical polyhydroxybutyrate (PHB) biosynthetic pathway has three basic steps catalyzed by three distinct enzymes: (1) condensation of two acetyl-CoA molecules forming one acetoacetyl-CoA catalyzed by *phaA* encoded β-ketothiolase, (2) reduction of acetoacetyl-CoA by *phaB* encoded NADPH-dependent acetoacetyl CoA dehydrogenase, and (3) polymerization of 3-hydroxyacid (3HA) units, i.e., (R)-3-hydroxybutyryl-CoA by *phaC* encoded PHA synthase. In addition to this basic pathway, 3-HA units can also be provided to PHA synthase (PhaC) by other secondary pathways involving methylmalonyl CoA pathway, *de novo* fatty acid synthetic pathway, and fatty acid β-oxidation pathway. As evident, PHA synthase plays a central and critical role in PHA biosynthesis (Singh et al., 2009, 2015). Heterotrophic bacteria are well studied and explained to have three classes of PHA synthase having specificity to substrate (3-HA) C-chain length. These include Class I PhaC (scl-3HA), Class II PhaC (mcl-3HA), Class III PhaEC with two subunits (EC) (scl-3HA), and Class IV PhaRC with two subunits (RC) (scl-3HA). Based on the presence of these enzymes, a wide range of substrates including sugars (glucose, fructose, sucrose, maltose, and lactose), starch, glycerol, FAs and its derivatives, methanol, lignin, agricultural, industrial, and dairy by-products have been reported as potential substrates for PHA production (Kumar et al., 2019; Bhatia et al., 2021; Kalia et al., 2021). With the addition of different substrates combinations, precursor substrates, adopting different feeding regime PHA compositions and content have been observed to improve (Kumar et al., 2013, 2014; Singh et al., 2015; Ray and Kalia, 2017). Figure 1 summarizes the PHA production by different bacteria on a wide range of substrates.

However, in cyanobacteria which can adapt for both photoautotrophic and heterotrophic cultivation, biosynthetic pathways for the utilization of exogenously provided C sources are more or less similar with the three basic steps discussed above. It is important to note that most of the information for PHA biosynthesis in cyanobacteria has been gained based on studies done on *Synechocystis* sp. PCC 6803. Here, synthase enzyme has two subunits PhaEC. Thus, PHA synthase in cyanobacteria belong to the type-III PHA synthases. Genes encoding the enzyme PhaA and PhaB are reported to be arranged in one operon and genes for PhaE and PhaC are arranged in one operon. Therefore, till now only SCL-PHAs are reported so far in the cyanobacterial species (Hai et al., 2001). Presence of a type-III PHA synthase has also been reported in *Synechococcus* sp. strains MA19 and PCC 6715, *Chlorogloeopsis fritschii* PCC 6912, *Anabaena cylindrica* SAG 1403-2, *Cyanothece* sp. strains PCC 7424, PCC 8303 and PCC 8801, and *Gloeocapsa* sp. strain PCC 7428. However, since cyanobacteria is a diverse group, the presence of other types of PHA synthase in them is still unclear and presents a great opportunity in near future (Troschl et al., 2017).

**FIGURE 2:**
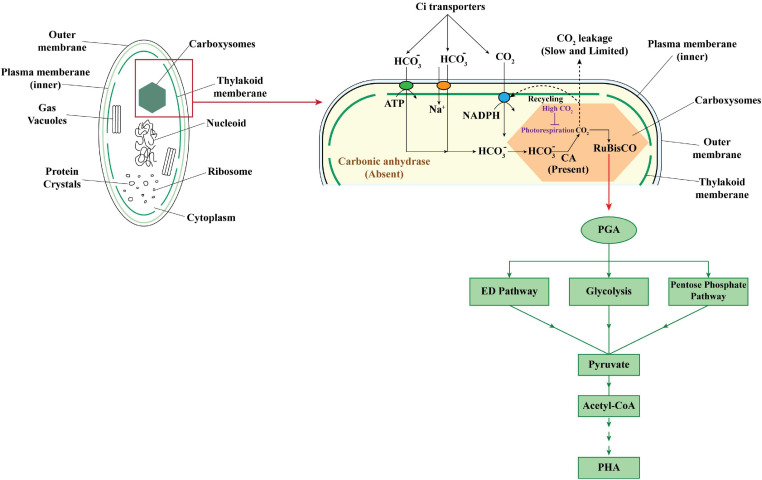
Carbon concentrating mechanism in cyanobacteria for CO_2_ fixation and further conversion to PHA.

While in autotrophic cultivation of cyanobacteria CO_2_, the C-source was fixed in Calvin–Benson–Bassham (CBB) cycle with the help of ribulose-1, 5-bisphosphate carboxylase/oxygenase (RuBisCO). RuBisCO has higher efficiency for CO_2_ than O_2_ and is known to be responsible for assimilation of 90% of the carbon present in biomass on earth. Atmospheric CO_2_ is transported across the wall with the help of inorganic carbon (Ci) transporters, thus helping to maintain local carbon concentration for RuBisCO. The output of the Calvin cycle, glyceraldehyde-3-phosphate after its conversion to 3-phosphoglycerate (PGA), can then enter into any of the three pathways for sugar metabolism, i.e., Entner–Doudoroff (ED) pathway, glycolysis, pentose phosphate pathway, and be finally converted to acetyl-CoA to be used in PHA synthetic pathway (Singh and Mallick, 2017; Figure 2).

## PHA Production in Cyanobacteria

A number of bacteria under this group are reported so far for PHA production majorly including the genus *Nostoc*, *Arthrospira*, *Synechocystis*, and *Synechococcu*. Here the maximum yield varied from 1 to 78% depending on the cultivation conditions (Table 1). Invariably, PHA production was seen under nitogen (N) or phosphorus (P) limitation and during late exponential or stationary phase.

**TABLE 1:** PHA production from cyanobacteria under different cultivation conditions (I): *Nostoc*, *Arthrospira*, *Synechocystis*, and *Synechococcus*.

**Organism**	**Stimulatory culture condition/substrate**	**Yield % (dry cell weight)**	**Bioproduct**	**References**
**CO_2_ as substrate: Photoautotrophic cultivation**				
*Nostoc*	Additional CO_2_	22	PHB	Haase et al., 2012
	Phosphate limitation	22.7	PHB	Panda et al., 2005
	-	19.27	PHA	Martins et al., 2017
	-	8.5	PHB	Sharma and Mallick, 2005a
*Arthrospira*	-	20.62	PHA	Martins et al., 2017
	Increased salinity	7.45	PHB	Shrivastav et al., 2010
	Phosphate limitation	3.5	PHB	Panda et al., 2005
	Nitrogen limitation	NA	PHB	Deschoenmaeker et al., 2016
*Synechocystis*	Nitrogen and phosphorous limitation	16.4	PHB	Kamravamanesh et al., 2017
	Nitrate limitation	12.5	PHB	Troschl et al., 2018
	Nitrogen and phosphorus limitation	11	PHB	Panda et al., 2006
	Nitrogen and phosphorus limitation	7.5	PHB	Wagner et al., 2016
	Photoautotrophic nitrogen limitation	4.1	PHB	Wu et al., 2001
	Nitrogen limitation	1	PHB	Carpine et al., 2015
*Synechococcus*	Phosphate limitation	55	PHB	Nishioka et al., 2001
**Exogenous substrates: Heterotrophic/mixotrophic cultivation**				
*Nostoc*	Acetate, glucose, valerate + nitrogen limitation	78	P(3HB-co-3HV)	Bhati and Mallick, 2015
	Mixotrophic with poultry litter	65	P(3HB-co-3HV)	Bhati and Mallick, 2016
	Acetate, and valerate + nitrogen and phosphorus limitation	60	P(3HB-co-3HV)	Bhati and Mallick, 2012
	Mixotrophic with glucose, acetate + gas exchange limitation	40	PHB	Sharma and Mallick, 2005b
	Acetate + glucose	40-43	PHB	Sharma and Mallick, 2005b
	Acetate, dark incubation after light-dark acclimatization	35	PHB	Sharma and Mallick, 2005a
	Acetate and propionate	31.4	P(3HB-*co*-3HV)	Mallick et al., 2007
	Acetate and glucose + phosphate limitation	16.6	PHB	Haase et al., 2012
	Acetate + glucose and dark incubation	45.6	PHB	Sharma et al., 2007
*Arthrospira*	Mixotrophic with acetate	3.0	PHB	De Philippis et al., 1992
*Synechocystis*	Fructose and acetate + phosphate and gas exchange limitation	38	PHB	Panda and Mallick, 2007
	Acetate + phosphate limitation	29	PHB	Panda et al., 2006
	Acetate + nitrogen limitation	7	PHB	Sudesh et al., 2001
	Digestate + mineral medium	6.6	PHA	Kovalcik et al., 2017
*Synechococcus*	Nitrogen limitation + dark incubation	27	PHB	Miyake et al., 1996

### Nostoc

*Nostoc* is a diverse group of terrestrial as well as aquatic filamentous cyanobacteria occurring around freshwater sources. Since the first report of PHB production that was 8.6% of cdw by *Nostoc muscorum* under photoautotrophic conditions in 2005, number of species have been reported for PHA production under phosphate and nitrogen limitation (Sharma and Mallick, 2005a). Later, PHB content up to 19.27% with *N*. *ellipsosporum* and 22% with *N*. *muscorum* was reported under photoautotrophic conditions (Panda et al., 2005; Martins et al., 2017). Diverting the flux toward PHB production pathway by using metabolic inhibitors carbonylcyanide *m*-chlorophenylhydrazone (CCCP) and dicyclohexylcarbodiimide (DCCD) resulted in PHB accumulation increment up to 21 and 17% from an initial PHB content of 8.5% of dry weight, respectively (Mallick et al., 2007). Sharma and Mallick (2005a, b) have reported the effect of culture conditions in improving the PHB production where it could be improved to 35% under mixotrophic condition with 0.4% glucose and acetate while a further improvement to 40–43% with gas exchange limitation under mixotrophy and chemoheterotrophy with 0.4% (w/v) acetate. Highest PHB production by *N. muscorum* was obtained up to 145.1 mg/L (16.6% of cdw) with 1% glucose and 1% acetate under phosphate starved conditions and CO_2_ supply (Haase et al., 2012). In addition to nutrient limitation and mixotrophy/heterotrophy, reduction in dark period, i.e., optimizing the light-dark cycle also, has a positive impact on PHB production as observed on *N. muscorum* using response surface methodology (Sharma et al., 2007).

It is interesting to note that PHA copolymer poly(3-hydroxybutyric acid-co-3-hydroxyvaleric acid), P(3HB-co-3HV), production has also been reported from *Nostoc* but that is possible only under mixotrophic or heterotrophic condition with supplementation of propionate or valerate in culture medium. Reports have shown P(3HB-co-3HV) production on addition of these precursor substrates up to 28.2–31.4% (Mallick et al., 2007) and 58–60% of cdw under nitrogen and phosphate limitation, respectively, which further improved to a highest content of 78% of cdw under heterotrophic conditions (Bhati and Mallick, 2012, 2015). Poultry litter was used as nutrient supplement at the rate 10 g/L in culture along with glucose, acetate, valerate, and CO_2_ and resulted in the production of P(3HB-co-3HV) copolymer yield of 774 mg L–1 (65–70% of dry cell wt.) (Bhati and Mallick, 2016). Notably, biomass achieved with *Nostoc* was not very high with yield around 1 g/L in most cases, and PHA accumulation in biomass was seen in late exponential phase.

### Arthrospira

*Arthrospira*, earlier also known as *Spirulina*, are a type of filamentous bacteria found in alkaline (salt) lake but can be grown in freshwater also. *Arthrospira* at present is mainly cultivated as food supplement due to its protein and vitamin rich content. But it is a strong candidate to be used for economical PHA production due to the fact that it shows high growth rate with less water requirement. As reported, *Arthrospira* can achieve 5000–15000 tons dry weight production per year (Spolaore et al., 2006; Lu et al., 2011). The requirement of high alkalinity for its cultivation renders the culture free from other common contaminants. As a result, it can very well be used to maintain a stable and high biomass culture in open ponds. *Spirulina platensis* is one of the commonly explored species for PHB production (Panda et al., 2005). Ever since the first report of PHB production in 1982 which was achieved up to 6% cdw, there has been no significant improvement in PHA production under photoautotrophic conditions. Different strategies have been employed to improve the yield. PHB yield improvement was seen with nitrogen starvation (Deschoenmaeker et al., 2016), phosphate limitation (3.5% of cdw) (Panda et al., 2005), acetate supplement (2.5–3% of cdw) (De Philippis et al., 1992) increased salinity (14.7%) (Shrivastav et al., 2010). Highest yields were obtained with *Spirulina* sp. LEB 18 which produced 1.48 g/L/d PHB in 15 d equivalent to 20.62% of cdw (Martins et al., 2017). As evident, improvement in the yield of PHA production is the major issue related to *Arthrospira* and Mixotrophic or heterotrophic culture, and condition optimization has helped and can help further to improve the yields.

### *Synechocystis* and *Synechococcus*

*Synechocystis* and *Synechococcus* are the genus of small-sized cyanobacteria found in both fresh and salt water. PHB production from *Synechocystis* was observed in 2001 where a yield of 4.1% was obtained under nitrogen-starved conditions (Wu et al., 2001). In this study, mixotrophic cultivation with glucose led to increase in biomass but not PHB yield (% of cdw). Here, the yield was improved to 9.5 and 15.2% of cdw under nitrogen limitation and 11.2% under phosphorus depletion in comparison to yield of 4.5% of cdw in balanced culture cultivation (Panda et al., 2006; Panda and Mallick, 2007; Wu et al., 2020). Samantaray et al. (2011) reported that heterotrophic cultivation with 0.4% acetate under dark incubation could improve the yield to 22% of cdw which was further improved to 38% of cdw when the stationary phase culture was subjected to phosphate and gas exchange limitation in the presence of acetate (0.4%) and fructose (0.4%) (Panda and Mallick, 2007). Till now *Synechocystis* PCC6803 is the most widely used strain for PHA production (Carpine et al., 2015). Closely related genus *Synechococcus* are also reported in literature for PHA production. *Synechococcus* sp. MA19, a thermophilic cyanobacterium which was a natural inhabitant of wet volcanic rock surface in Japan, produced PHB utilizing CO_2_ up to 27% (w/w) (Miyake et al., 1996). The yields from this strain in photoautotrophic cultivation was further improved to 55% (dcw) amounting to 2.4 g/L under phosphate limitation. This is the highest PHA production reported so far from cyanobacteria under photoautotrophic conditions (Nishioka et al., 2001). *S. salina* was also reported to produce 7.5% of PHB which was further used after hydrothermal liquefaction for bio-oil production (Wagner et al., 2016). In another report it produced comparable yields of 4.8–9% of cdw reaching up to 2.0 g/L in 21 days of incubation in a large tubular photobioreactor with medium circulated, pH controlled through CO_2_ flow, artificial illumination, and controlled temperature. Here, nitrogen limitation and illumination were observed to improve the PHA production while mixotrophy by the addition of acetate did not help due to contamination (Kovalcik et al., 2017). There has been not much reports for PHA production by *Synechococcus* sp., and hence, it leaves a lot of scope to try mixotrophic or heterotrophic cultivation to improve the production.

In addition to the above-mentioned genus that are majorly used for PHA production, other genus have also been reported to have PHA producing abilities. These genera include *Chlorogloea fritschii*, *Gloeocapsa* sp., *Oscillatoria limosa*, *Gloeothece* sp. PCC 6909, *Aulosira* sp., and *Calothrix* (Stal et al., 1990; Hai et al., 2001; Samantaray et al., 2011; Kaewbai-ngam et al., 2016; Table 2). *Chlorogloeopsis fritschii* PCC 6912, which is a thermophilic cyanobacteria, was reported to have 6% of PHB in its dried biomass (Hai et al., 2001), while *Brevibacillus invocatus* MTCC 9039 had PHB up to 3% of cdw accumulated in its stationary phase (Sankhla et al., 2010). Samantaray and Mallick (2012) reported the production of PHB simultaneously along with wastewater treatment in a biological recirculatory system by N_2_-fixing cyanobacterium *Aulosira fertilissima.* This bacteria was also reported to produce 77% PHB under phosphate deficiency with 0.5% acetate and improved to 85% of cdw of PHB with 0.26% citrate, 0.28% acetate, and 5.58 mg/L K_2_HPO_4_ for an incubation period of 5 days (Samantaray et al., 2011). Similarly under nitrogen limitation, *Calothrix scytonemicola* TISTR 8095 could produce 25% of cdw (356.6 mg/L) PHB in 44 days (Kaewbai-ngam et al., 2016). *Anabaena cylindrica*, a filamentous cyanobacterium, could produce 2% of cdw PHB while P(3HB-co-3HV) was produced on supplementation of valerate and propionate (Lama et al., 1996).

**TABLE 2:** PHA production from cyanobacteria under different cultivation conditions (II).

**Organism**	**Substrate and culture condition**	**Yield % (dcw)**	**Bioproduct**	**References**
**CO_2_ as substrate: Photoautotrophic cultivation**				
*Aulosira fertilissima*	CO_2_ + waste water	34.8 (41 g/m^2^)	PHB	Samantaray et al., 2011
*Calothrix scytonemicola*	Nitrogen lim.	25.2	PHB	Kaewbai-ngam et al., 2016
*Anabaena* sp.	-	2.3	PHB	Gopi et al., 2014
*Phormidium*	-	2.3	PHB	Gopi et al., 2014
**Exogenous substrates: Heterotrophic/mixotrophic cultivation**				
*Aulosira fertilissima*	Waste water + citrate + acetate	87.22 (92 g/m^2^)	PHB	Samantaray et al., 2011
	Acetate + phosphorous limitation	77	PHB	Samantaray and Mallick, 2012
	Fructose + valerate supplementation/phosphate deficiency	62-77	P(3HB-co-3HV)	Samantaray and Mallick, 2014
	Acetate + citrate	66	PHB	Samantaray and Mallick, 2012
	Citrate + phosphate limitation + dark	51	PHB	Samantaray and Mallick, 2012
*Chlorogloeopsis fritschii*	Mixotrophic with acetate	6.2	PHB	Hai et al., 2001
*Scytonema geitleri*	Acetate	7.12	PHB	Singh et al., 2019
*Anabaena cylindrica*	Acetate	2	PHB	Lama et al., 1996
	Acetate + propionate		PHB	Lama et al., 1996

### Recombinant Cyanobacteria

Genetic engineering to modify microorganisms toward specific needs or property has been a well exploited practice in case of prokaryotic research and development. Since prokaryotes are relatively easy to manipulate due to simple genetic makeup in comparison to algae and plants, cyanobacteria also possess such attributes. Spontaneous transformability and short generation time have made *Synechocystis* sp. PCC6803 as the best studied model cyanobacterium not only for PHA production but other cyanobacteria related researches too. Moreover, *Synechocystis* sp. PCC6803 also has the honor of being the first photoautotrophic organism to have the complete genome sequenced in 1997 and first recombinant cyanobacteria used for PHA production (Wilde and Dienst, 2011; Troschl et al., 2017; Kamravamanesh et al., 2018a,b). As observed in studies discussed above, recombinant cyanobacteria also showed improvement with nutrient limitation and mixotrophic cultivation (Table 3). PHA biosynthesis can be improved by introducing multicopies of heterologous PHA synthase gene up to 11 wt% of the cdw (Sudesh et al., 2001). The ADP-glucose pyrophosphorylase gene (agp) was inserted with an erythromycin resistance cassette and under nitrogen depletion, photoautotrophic PHB production was up to 14.6% which improved under mixotrophic cultivation with acetate up to 18.6% (Wu et al., 2002). Inactivation of Thesll0783 gene by of kanamycin/bleomycin resistance cassettes and cultivation under nitrogen depletion indicated the importance of Thesll0783 gene in PHB production (Schlebusch and Forchhammer, 2010). The native SigE gene was overexpressed into *Synechocystis*, to control changes in sugar catabolism related to glycogen pathway which resulted in 1.4% of cdw phototrophically (Osanai et al., 2013). Similarly, overexpression of PHA biosynthetic operon from *Microcystis aeruginosa* NIES-843 in *Synechocystis*, resulted in 7% of cdw photoautotrophic PHB production under nitrogen depletion (Hondo et al., 2015). In another report, out of the four native genes chosen for overexpression (*phaAB*, *phaEC*, and *phaABEC*), maximum PHB content of 26% of cdw was achieved under nitrogen depletion with *phaAB* overexpression as compared to 9.5% of cdw in wild-type which further improved to 35% under mixotrophic conditions with supplementation of 0.4% acetate under nitrogen depletion (Khetkorn et al., 2016). Although not PHA, Wang et al. (2013) have reported 3-hydroxybutyrate by phaEC inactivated *Synechocystis* having heterologous expression of thioesterase gene (*tesB*) from *E. coli* and *phaAB* gene from *Cupriavidus necator* H16 under photoautotrophic utilizing CO_2_. Overexpression of PHA synthase from *Chromobacterium* sp. USM2 (*phaC*_*C*__*s*_), acetoacetyl-CoA synthase from *Streptomyces* sp. CL190 (*nphT7*_*S*__*s*_), and *C*. *necator* acetoacetyl-CoA reductase (*phaB*_*Cn*_) genes under the control of the light-inducible *psbAII* promoter under direct photosynthesis produced 14% cdw yield, among the best, under photoautotrophic cultivation and were observed to further improve to 41% on addition of acetate 0.4% (Lau et al., 2014). Recently, a strategy of increasing acetyl-CoA level by inducing deletions of phosphotransacetylase (Pta) and acetyl-CoA hydrolase (Ach) and the expression of a heterologous phosphoketolase (XfpK) from *Bifidobacterium breve* with CO_2_ as feed produced 12% of cdw (232 mg/L) (Carpine et al., 2017). Optimization of acetyl CoA reductase binding site with CO_2_ produced 1.84 g/L PHB with productivity of 263 mg/L/d (Wang et al., 2018).

**TABLE 3:** PHA production from recombinant cyanobacteria under different cultivation conditions.

**Organism**	**Recombinant factor/Culture media**	**Yield % (dry cell weight)**	**Biopolymer**	**References**
**CO_2_ as substrate: Photoautotrophic cultivation**				
*Synechocystis*	Random UV mutagenesis	37	PHB	Kamravamanesh et al., 2018a
	Optimized ribosome binding site for *phaB1*	35	PHB	Wang et al., 2018
	Overexpression of native *phaA* and *phaB* Nitrogen deprivation	26	PHB	Khetkorn et al., 2016
	Overexpression of the sigma factor *sigE* Nitrogen limitation	25	PHB	Osanai et al., 2013
	*agp* gene—deletion-mutant	14.9	PHB	Wu et al., 2002
	Overexpression *phaC*_*C*__*s*_, *nphT7*_*S*__*s*_, and *phaB*_*Cn*_ under the control of the *psbAII* promoter Increased CO_2_	14–16%	PHB	Lau et al., 2014
	*xfpk* overexpression in double *pta* and *ach* mutant	12	PHB	Carpine et al., 2017
	Overexpression of PHA biosynthetic operon from *Microcystis aeruginosa*	7.0	PHB	Hondo et al., 2015
	Knockout mutant of the sll0783 gene Nitrogen limitation	NA	PHB	Schlebusch and Forchhammer, 2010
*Synechococcus*	*Pha genes in complemented* cyanobacterial *recA* null mutant with the *E. coli recA* Nitrogen limitation	52	PHB	Akiyama et al., 2011
	PHA biosynthesis genes from C. necator	1.85	PHB	Takahashi et al., 1998
**Exogenous substrates: Heterotrophic/mixotrophic cultivation**				
*Synechocystis*	Overexpression *phaC*_*C*__*s*_, *nphT7*_*S*__*s*_ and *phaB*_*Cn*_ under the control of the *psbAII* promoter Acetate + gas exchange limitation	41	PHB	Lau et al., 2014
	Overexpression of native *phaA* and *phaB* Acetate and nitrogen Limitation	35	PHB	Khetkorn et al., 2016
	*agp* gene—deletion-mutant Acetate	18.6	PHB	Wu et al., 2002
	PHA biosynthetic genes of *C. necator* Acetate and nitrogen limitation	11	PHB	Sudesh et al., 2002
	PHA biosynthetic operon from *C. necator* Acetate and nitrogen limitation	11	PHB	Sudesh et al., 2001
*Synechococcus*	PHA biosynthesis genes from *C. necator* Acetate and nitrogen limitation	26	PHB	Takahashi et al., 1998

Genetic manipulations have also been successfully employed in *Synechococcus*. *Synechococcus* sp. PCC7942 having PHB synthesizing genes from *Alcaligenes eutrophus* produced 3.01% of cdw PHB under photoautotrophic and nitrogen-starved conditions which was further improved 25.6% of cdw on supplementation of acetate under nitrogen-starvation (Takahashi et al., 1998). Recombinant cyanobacteria *Synechococcus* sp. PCC7002 *recA* null mutation with the *E. coli* recA gene achieved 52% of cdw PHA from carbon dioxide in Antibiotics-free cultivation (Akiyama et al., 2011). *Synechococcus PCC7942* having heterologous expression of *C. necator* PHA operon showed improved PHA accumulation, i.e., from 3 to 25% of cdw (Drosg et al., 2015) and introduction of GABA shunt with CO_2_ feed led to PHA yield of 4.5% cdw (Zhang et al., 2016). In an interesting report, polymer with 4-hydroxybutyrate (4HB) monomer also, i.e., copolymer P(3HB-co-4HB) was produced with the introduction of PHA biosynthetic genes from *Chlorogloeopsis fritschii* PCC 9212 and *ccmR* gene deletion in *Synechococcus* sp. PCC 7002. This study provided PHA content of ∼4.5% of cdw and was an indication of possibility of P(3HB-co-4HB) production in cyanobacteria using light and CO_2_ (Zhang et al., 2015).

## Hybrid Biological System

Although the PHA production in heterotrophic cultivation have attained a significant level as far as the yield per unit feed are concerned. However, still the feed procurement cost is one of the major concerns making it an expensive practice to follow. As discussed till now, it is quite clear that due to the high photosynthetic efficiency, minimal nutrient requirement, high growth per hectare and CO_2_ sequestering ability, cyanobacteria has gained the attention of researchers in biotechnology across the globe. In addition to that other properties such as no specific agricultural land requirement as compared to plants, efficient waste water utilization are also notably interesting. Possibilities of genetic engineering have added to its popularity providing a novel low cost expression system. Thus, PHA production from cyanobacteria eliminates the factors causing high production cost, i.e., exogenous C substrates, nutrient maintenance, etc. But, as with any other system photoautotrophic PHA production also has its limitations which need to be addressed while developing a sustainable system. It is quite evident that photoautotrophic cultivation in laboratory photobioreactors are helpful in maintaining monoculture but need continuous illumination which requires high maintenance, thus causing high production cost. In comparison, open pond cultures are economical but maintaining a contamination free monoculture is a challenge. Moreover, the uncertainty due to fluctuation in temperature, pH, light intensity, and CO_2_ fixation caused by diurnal and seasonal changes leads to less efficient system (Kamravamanesh et al., 2018b).

Thus, the need of the hour is to develop a hybrid biological system that can address the challenges and utilizes the opportunities present in both photoautotrophic and heterotrophic cultivation. Various reports have shown that PHA during photoautotrophic cultivation is lower and can be achieved up to 55% only in *Synechococcus* sp. (Nishioka et al., 2001). Thus, here we suggest the following cultivation regimes and strategies to improve PHA production.

### Mixotrophy

**FIGURE 3:**
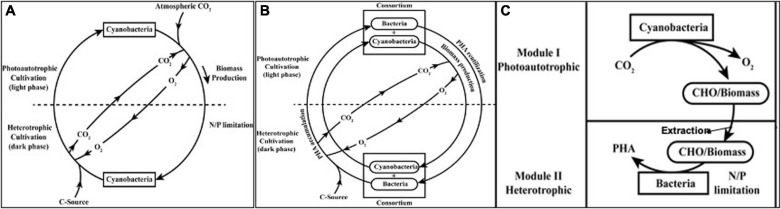
Strategies and culture regime of “hybrid biological system” for PHA production improvement. **(A)** Mixotrophy; **(B)** Photoautotrophic-heterotrophic consortium; and **(C)** Two module system.

Invariably in all the cases reported so far mixotrophic cultivation or heterotrophic cultivation have resulted in significant improvement in PHA yields (Sudesh et al., 2002; Sharma and Mallick, 2005b; Panda et al., 2006; Wagner et al., 2016). There has been 2–9-fold improvement in PHA yield (% of cdw). This suggests a possibility to use photoautotrophic and heterotrophic component (cultivation) in same system. The proposed system having a cyanobacteria as PHA producer may be subjected to photoautotrophic cultivation during day time/light phase followed by the introduction of heterotrophic cultivation during night time/dark phase (Hai et al., 2001; Sharma and Mallick, 2005b; Sharma et al., 2007; Bhati and Mallick, 2016; Kovalcik et al., 2017). During light phase, cyanobacteria will utilize CO_2_ as carbon source, and in dark phase, exogenous C sources (glucose, acetate, copolymer precursors, etc.) may be added in media. An alternative improvement takes high biomass growth and lower PHA yield in cyanobacteria under photoautotrophic cultivation under consideration. In this system (Figure 3A), cyanobacteria may be subjected to photoautotrophic conditions during day time/light phase without any N/P limitation to attain high biomass till the late exponential or stationary phase and then subjected to heterotrophic conditions in night time/dark phase under N/P limitations. This may constitute a feast (dark) and famine (light) phase, promoting PHA production in microorganisms.

### Photoautotrophic-Heterotrophic Consortium

The main aim of developing a consortium is to have a more efficient system through the division of labor that gives enhanced accessibility of resources, enhanced productivity, efficient nutrient cycling, community stability and non-competitive partitioning, and distribution of carbon or energy source between members of community based on metabolic functionality. Out of the different interaction aspects, mutualistic relationship is the most widely exploited for numerous biotechnology studies such as bioprocess, biofuel, and other value-added products formation (Bernstein and Carlson, 2012). Photosynthetic consortium having algae and bacteria has been reported for PHA production. Here, PHA production was obtained up to 20–30% of cdw without oxygen supplementation. In this study the consortium was subjected to feast and famine regime where the PHA production was achieved in feast phase during dark cultivation. During famine phase in illuminated cultivation, accumulated PHA can be oxidized and consumed releasing the reducing equivalents and CO_2_. The reducing equivalents produced during this famine phase are used in PHA production process in feast phase, while the CO_2_ released is used by algae for photosynthesis. During photosynthesis, algae released O_2_ which reduced the aeration requirement and was also used for PHA oxidation. Thus, this study provided an evidence of efficient resource utilization by consortium (Fradinho et al., 2013a,b). Another consortium of microalga *Chlorella vulgaris* and the bacterium *Pseudomonas aeruginosa* was developed by Guerra-Renteria et al. (2019) for nitrogen and phosphorus uptake from wastewater. Thus the consortium can be used for organic waste degradation also where oxygen release from algae is utilized by bacteria to degrade organic matter simultaneously CO_2_ release from the bacteria is utilized by microalgae as raw material to complete photosynthesis. This symbiotic interaction helps to reduce wastewater treatment efforts by natural way. A recent study has reported lichen-associated *Pseudomonas* having the ability to detoxify and utilize naphthalene and anthracene for energy reserve storage and produce PHA with 3-HHx unit up to 30.62 and 19.63% of 3-HHx, respectively (Nahar et al., 2019). The proposed system has consortium of microalgae/cyanobacteria and bacteria as two components so as to utilize resources under both light and dark phase (Figure 3B).

### Two Module System

Since the PHA yield in fast growing photoautotrophic cultivation, the PHA yields are low. Thus, either the high biomass or the organic products released by them can alternatively be used as substrate itself. Hydrothermal liquefaction (HTL) is the efficient strategy to obtain organic compounds from cyanobacterial biomass, e.g., propylene and bio oil (biofuel) production was shown from cyanobacterial biomass processed through HTL. This suggested PHB as a feedstock for biorefinery as propylene level was found to be proportional to the accumulated amount of PHB (Wagner et al., 2016). One of the study revealed that microalgae grown in wastewater can be harvested using different methods. The harvested biomass can further be used as a substrate for growth of genetically engineered *E. coli* and PHB production (Rahman et al., 2014). Using defatted biomass also gives an attractive option as reported by Goo et al. He showed 82% biopolymer yield using defatted mass of microalgae *Dunaliella tertiolecta* with different salt concentration (Goo et al., 2013). In a recent report, PHA production was reported in two modules with synthetic mixed culture of *Synechococcus elongatus cscB* and *Pseudomonas putida* cscAB. Here in the first module, *Synechococcus elongatus* cscB was cultivated under photoautotrophic condition where it fixes CO_2_ to convert it into sucrose and release it into culture medium. In the second module, *Pseudomonas putida* csc AB was used under heterotrophic cultivation to utilize sucrose produced in first module for PHA production under nitrogen limitation (Lowe et al., 2017). The proposed system, depicted in Figure 3C, may have photoautotrophic cyanobacterial growth in one module, and then in second module after the processing of its biomass, it can be utilized by heterotrophic bacteria as substrate for PHA production.

### Metabolic Network Modeling

A number of factors and parameters have been reported in literature those are affecting the growth and PHA production in microalgae. Some of them are temperature, light intensity, pH, salinity, CO_2_ exchange level, nitrogen level, phosphorous level etc. These factors are more detrimental when operating in a large scale or open pond culture. Even if, we use the above proposed strategies involving the use of consortium or two module system, optimization of these factors and additionally the metabolic interaction of member microorganisms, is a critical step and requires lot of efforts and resource utilization affecting the final performance and efficiency of any bioprocess. In the effort of developing a bioprocess for PHA production we need to have an insight into the enzymatic and metabolic pathways of the PHB biosynthesis. We need to select or manipulate genetically an optimal strain with maximum productivity. In addition to these, other requirements like the selection of inexpensive substrate, culture condition optimization of media, process parameter optimization for large scale production and designing a bioreactor ought to be managed carefully (Troschl et al., 2017). In present research world, mathematical or *in silico* tools are proving to be a boon in such studies, as these not only can handle integration and combination of large number of parameters or interactive connections to be analyzed but also reduce the time taken for experimental exercises to completely analyze that much number of combinations. Microbial consortia engineering through studying the highly complex interactions between populations and also with environment is increasingly been accepted to have potential to construct sustainable bioprocesses with enhanced metabolic productivity. Mathematical models including differential equations and stochastic methods have been used in microbial ecology modeling (Bernstein and Carlson, 2012). A number of mathematical models used in different studies for PHA production include kinetic, dynamic, cybernetic models (Dias et al., 2008; Jiang et al., 2011; Novak et al., 2015). Flux balance analysis (FBA) or in other words linear programming (LP) based on objective function and constraints is used in multiple studies, e.g., to predict the relative abundance of *Desulfovibrio vulgaris* (sulfate reducing bacteria) and *Methanococcus maripaludis* (methanogen) based on the analysis of mutual metabolic exchange between them (Stolyar et al., 2007), dynamic modeling for simulation of two different co-cultures for synergistic co-fermentation of xylose + glucose mixture with ethanol (Hanly and Henson, 2011, 2013), synthetic or semisynthetic co-cultures of *E. coli* analysis for xylose, and glucose utilization in *S. cerevisiae*. Another stoichiometric model is the elementary flux mode analysis (EFMA) that includes metabolic pathway network modeling and analysis (Volkova et al., 2020). EFMA has been used to study mass and energy flows through microbial community of a phototrophic, biofilm community (Taffs et al., 2009).

Somehow, majority of mathematical tools are not easy to use for non-mathematical background people. Thus, network-based modeling is a convenient option in such case. Metabolic network modeling-guided strains’ design or development is gaining interest and has been used for n-butanol, 1,3-propanediol, glycerol, limolene, and isoprene (Hendry et al., 2020). Genome-scale metabolic network reconstructions and constraint-based analyses have been used in number of studies for metabolic modeling (Lee et al., 2020). Similar strategy was used to predict and design a strain that has a force C-flux toward malonyl-CoA for enhanced production of polyketides and biofuels (Xu et al., 2011). Recently, a novel strain design algorithm OptRAM (Optimization of Regulatory and Metabolic Networks) was developed with integrative regulatory and metabolic network modeling. This tool has helped the researchers to identify and design strain for Succinate 2,3-butanediol and ethanol production in yeast (Shen et al., 2019). Similarly, mathematical and metabolic engineering were used for PHA production and recovery process improvement (Novak et al., 2015; Pillai et al., 2019). In another study, analysis and prediction of metabolic flexibility of *Candidatus Accumulibacter phosphatis*, a phosphate accumulating organism, was done based on redox factor preferences of different oxidoreductase (da Silva et al., 2020).

For network-based modeling, here we propose another tool Petri Net for such analysis. Petri Nets (PN) are directed bipartite graph in with vertices as two disjointed sets, i.e., places and transitions. Arcs connect vertices, i.e., transitions with places and places with transitions. The distribution of tokens, i.e., quantities of particular components over the places corresponds to the state of the modeled system. In biological representation “places” (circles) correspond to biological or chemical components, “Transitions” (rectangles) to processes (e.g., reactions) (Kansal et al., 2015). A transition is active only under certain dynamics and firing rules causing the flow of tokens, i.e., information and tokens are consumed from the input places through the transitions. PN provide a competent method for both qualitative (structural topology) and quantitative analysis (token distribution). Snoopy, MARCIE, WoPeD, and Pathway Logic Assistant are some of the tools used for petri net modeling in computational biology (Bartocci and Lio, 2016). Petri Net has the advantage of graphical representation of complex networks which is compatible to computational simulations and is user friendly to a non-mathematical population (Koch, 2015). It has been used to model various biological pathways/processes, e.g., metabolism, signal transduction, gene regulation, protein complex assembly, metabolic disorders for biomedical data prediction, and enzyme kinetic modeling (Koch et al., 2017). Recently, PN are used for modeling of biological pathways (PHA; C3 pathway), networks for processes involved in diagnostics, treatment, and multi drug resistance in tuberculosis (Gupta et al., 2019; Singh and Jha, 2019; Singh et al., 2020).

## Conclusion

In spite of having an advantage associated with photoautotrophic metabolism that eliminates the cost attributed to C substrate, cyanobacteria still have lower yield of photoautotrophic PHA production. However, still a sustainable biological system can be constructed if we can efficiently use the abilities and attributes related to photoautotrophic and heterotrophic PHA production. The proposed hybrid biological system involving mixotrophy, photoautotrophic–heterotrophic consortium and two module system along with the optimization of process parameters, and utilization of metabolic network modeling can achieve the required sustainability. Such biological system can provide an added advantage, as it not only has economical production of PHA but due to CO_2_ sequestering capabilities can help in reducing global warming effect and can reduce pollution by providing alternative of synthetic plastic.

## Author Contributions

RA, ST, and MS have contributed toward conceptualization and framing the review content for PHA production from microbes. GS has contributed for the conceptualization and integration of mathematical/metabolic model aspect. All authors contributed to the article and approved the submitted version.

## Conflict of Interest

The authors declare that the research was conducted in the absence of any commercial or financial relationships that could be construed as a potential conflict of interest.
